# Modern Polymers for Dental Application

**DOI:** 10.3390/biomedicines12020252

**Published:** 2024-01-23

**Authors:** Oliver Schierz

**Affiliations:** Department of Prosthetic Dentistry and Material Sciences, University of Leipzig, Liebigstr. 12, 04107 Leipzig, Germany; oliver.schierz@medizin.uni-leipzig.de

## 1. Introduction

Ceramics dominate clinical procedures in modern dentistry related to the artificial replacement of teeth with fixed dental prostheses, replacing metal-based frameworks on a large scale. In smaller cavities, direct composite restorations perform similarly to indirect restorations and are therefore more cost-effective [1,2]. However, for single tooth restorations, especially partial, full and implant-borne crowns, polymers now offer a routinely available time- and cost-effective alternative [3]. Nevertheless, there are two competing concepts under long-term observation, polymer-infused ceramic networks (PICNs) and highly nanofilled composites. Although polymers/dental composites have many applications in dentistry, they still face several problems, mainly the occurrence of secondary caries, restoration fractures, excessive wear and marginal degradation [4,5]. Extensive efforts are therefore being made to improve the compositions and microstructures of these composites and enhance their clinical performance and longevity. While current composites are generally bioinert and can replace the missing tooth structure, future composites should be bioactive and therapeutic to inhibit caries, modulate biofilms, and protect the surrounding tooth structure in order to increase the longevity of the restoration [6].

In the field of removable dental prostheses, metal frameworks still dominate daily clinical practice. However, the base materials of dentures are mainly made of methacrylate (MA)-based polymers. Despite the rapid development of polymer science, dental materials science has lagged behind in the utilization of these advanced polymer products. These polymers offer unique properties that are superior to traditional polymers and, more importantly, a range of properties that more closely resemble natural biomaterials. This allows their application in patients with certain intolerances to MA-based materials or indications that can only be achieved through special material properties, such as tooth-coloured splints and metal-free dentures; however, their use is limited to these special indications due to their limited flexural and fracture strengths and various chemical interactions [7,8]. Therefore, advancements in the application of polymers in dentistry should be vigorously pursued. In this Special Issue, we show a number of promising examples of how the latest generation of advanced polymers will improve the application of materials in dental clinics.

New materials for dental applications have become available in recent years through computer-aided manufacturing processes such as milling and 3D printing. Typical examples include dental veneers, partial crowns, splints and frameworks for fixed and removable dentures. However, there is no single polymer or composite material that is adequate for all indications or suitable for all manufacturing techniques. Most materials, in terms of their material properties, fall into either the fixed or the removable category.

MA-based resins remain the dominant choice in dentistry. This is probably due to the favourable thermophysical and chemical properties of MA-based resins. Other material options, such as nylon, polyoxymethylene or polyaryletherketone, play a minor role and are mainly used for temporary restorations or in patients with (potential) adverse reactions [9]. This situation is reflected in the research articles included in this Special Issue. However, new materials—known from tissue engineering—are emerging and being tested for their potential use in dental applications. For example, bioactive glass-based photocurable resins are among the most promising materials, exhibiting promising properties [10]. This Special Issue has collected a variety of studies focusing on polymers and composites in dental applications. It contains ten original research articles and one review, which I briefly describe in the following paragraphs. In doing so, I would like to clarify that it is not the purpose of this editorial to discuss each of the texts in detail, but rather to encourage the reader to explore them.

## 2. An Overview of Published Articles

Another noticeable trend in dentistry materials is the shift in manufacturing techniques. In this context, traditional thermo-polymerized MA-based resins serve as a control. Current research emphasizes CAD/CAM processes involving milling and printing. In addition, related CAD/CAM materials are a topic of increasing research interest.

In fixed restorations, the focus is mainly on milled MA-based composites that are highly filled with inorganic materials. However, the 3D printing of permanent composite crowns is already feasible and in clinical use. Therefore, some articles in this Special Issue focus on milled fixed prostheses. Since dental ceramics are the most popular alternative, they serve as a control. Studying the wear behaviour of these materials will increase our clinical knowledge and replace guesswork. Polymer-infiltrated ceramic networks (PICNs) are an interesting alternative to the more classical nanoresin-based millable composites. In contrast to most ceramics, resin-based systems can be applied without additional treatment and therefore show great potential in cost- and time-efficient applications. A tribological thesis often formed by clinicians is that the harder the material, the more intense the wear on antagonistic teeth. However, is this true?

Baldi’s article (Contribution 1) aimed to evaluate the wear rate of PICNs, composites and ceramics against enamel in a bruxism-simulating scenario using molars. The tests were carried out using a chewing simulator set at an 80 N load and semicircular motion in order to simulate bruxist movements and loads. The nanohybrid resin-based composite caused the least wear on the antagonist teeth but was itself worn at a rate comparable to enamel. In contrast, zirconia itself was wear-resistant, but caused slightly more wear to enamel. However, lithium silicate, one of the most popular ceramic materials for single crowns, caused the most extensive damage to healthy enamel [11].

The research by Chen et al. (Contribution 2) deals with the additive manufacturing of resin-based photocurable bioactive glass (BAG) scaffolds with high filler loading. The influence of the BAG content on the rheology of the resin and the rate of the polymerisation reaction was investigated, and suitable compositions for the stereolithographic fabrication of green bodies were described. This study’s results will be useful for preparing high-density glass/ceramic slurries and optimising their printing properties.

Graf’s work (Contribution 3) compared the adhesive properties of a 3D-printed and milled composite and a PICN in vitro using pull-off testing until retention loss was observed. No material-specific failure modes were detected and all clinical requirements were met.

Rosentritt et al. (Contribution 4) investigated eleven different resin-based composites and resin-infiltrated ceramics produced by milling. The aim of this investigation was to determine the effects of storage time on the properties of resin-based materials. A variety of thermophysical analyses were used in the study to gain insight into the composition of the resin-based materials and the influence of internal plasticisation and water sorption. For the milled resin-based composites, storage in water induced different degradation, heat energy and mechanical behaviours. However, some composites showed less influence from water storage and retained good mechanical properties.

Schmohl et al. (Contribution 5) looked into long-term resistance of five milled resin-based composites to three acidic media using demineralised water as a control. Changes in surface roughness and in-surface hardness were measured, and the damage mechanisms were analysed using sophisticated methods. Scanning electron microscopy revealed the leaching of barium, aluminium and titanium from fillers on the rough but not the polished surface of the samples.

For removable dentures, milling large objects is very time-consuming and is therefore a costly option. In addition, subtractive manufacturing is associated with significant material waste, which runs counter to the goal of resource-efficient materials. An alternative approach—3D printing—solves this contradiction. Printable materials have many limitations but have proven to be a versatile and therefore promising technology. As a result, the properties of the available printable materials are being intensively investigated. Fittingly, this Special Issue includes an article showing the influence of graphene modification on antimicrobial activity. Differences in surface roughness due to different polishing protocols as well as mechanical properties are also topics worth investigating. Therefore, the following articles address these topics from different angles.

The sixth paper published in this Special Issue is a systematic review by Lourinho et al. (Contribution 6) on the mechanical properties of conventionally polymerised and 3D-printed polymethyl methacrylate (PMMA) using the PRISMA guidelines. Flexural strength data were extracted from eight articles and included in a meta-analysis. The 3D-printed resin still showed lower flexural strength and hardness compared to the thermoset resin. In contrast, the thermoset resin showed less favourable results for impact strength.

Quezada et al. (Contribution 7) investigated the surface roughness (Ra) of denture base acrylic resins obtained through different processing techniques and using the same polishing instruments. They also compared a manual polishing technique with a prototype for mechanised polishing. However, the results were not consistent between the two techniques. While manual polishing revealed significant differences between milled and 3D-printed resin, and milled and thermoset resin, the mechanised technique revealed differences between the self-cured and 3D-printed resin and self-cured and thermoset resin.

The study by Salgado (Contribution 8) tested the antimicrobial activity of a 3D-printed graphene-enhanced acrylate resin against Candida albicans and Streptococcus mutans in vitro in addition to the material’s surface roughness using four different concentrations of graphene and neat resin as a control. The surface roughness increased with the increasing graphene concentration. The surface adhesion showed that the density of microbial biofilms decreased in samples doped with graphene for at least 48 h. Therefore, despite increasing the surface roughness of the resin, graphene has anti-adhesive effects against the two main microorganisms responsible for prosthetic stomatitis [12].

The behaviour of direct restoratives is still of interest in special situations such as patients with leukaemia or bulimia patients (intense acid contact).

In the article by Mester et al. (Contribution 9), four resin-based composites were tested in vitro by immersing them in the saliva of leukaemia patients before the start of chemotherapy regiments and in controls. Water sorption, water solubility and residual monomers were tested. The behaviour of the samples in different immersion environments varied according to the filler ratio of the composite and the types of organic matrix and filler. The solubility was inversely proportional to the sorption values. The highest amounts of residual monomers were found in the saliva of leukaemia patients. However, the differences found were small.

Nica et al. (Contribution 10) presented a study comparing the surface roughness of a conventional and a resin-modified glass ionomer cement after different immersion times in some acidic beverages. For both materials, the highest surface roughness was found after 14 days of immersion in acidic beverages. The traditional cement was more affected by the acidic environment compared to resin-modified cement.

Taraboanta et al. (Contribution 11) investigated the short-term resistance of three bulk-fill resin-based composites to hydrochloric acid and then to the abrasive effect of one year of toothbrushing at different times. The short-tern exposure to hydrochloric acid had no relevant effect on the surface roughness of the bulk-fill composite resins.

## 3. Conclusions

This compilation of articles on dental polymers covers a wide range of research, illustrating the richness of this field. This richness is reflected in the different methodologies used in the studies, ranging from quantitative approaches based on in vitro observations to reviews that combine the available information to summarize the knowledge gathered. Most of the papers in the Special Issue deal with materials for fixed dental prostheses, many of which are already in daily clinical use. In contrast, resins for removable dental prostheses and direct restoratives were less represented. Finally, I would also like to point out the peculiarity that all the texts are based on studies from European countries.
